# The relationship between body pain, physical fatigue, and mental fatigue in Chinese adolescents

**DOI:** 10.3389/fpubh.2026.1828676

**Published:** 2026-06-05

**Authors:** Xinzhu Wang

**Affiliations:** School of Teacher Education, Xichang College, Xichang, China

**Keywords:** body pain, Chinese adolescents, gender, mental fatigue, physical fatigue

## Abstract

**Background:**

Body pain and fatigue are significant components of subhealth in adolescents. However, few studies to date have examined the cross-temporal interrelationships among body pain, physical fatigue, and mental fatigue in Chinese adolescents.

**Methods:**

This study employed a longitudinal design. Body pain, physical fatigue, and mental fatigue were assessed in 966 Chinese adolescents through two waves of surveys conducted 1 year apart. Descriptive statistics, Harman’s single-factor test, Pearson correlation, and structural equation modeling were used to analyze the data.

**Results:**

Among Chinese adolescents, body pain and physical fatigue mutually predict each other over a one-year interval, and physical fatigue and mental fatigue also exhibit a bidirectional predictive relationship over time, with all effects reaching a medium level. However, the mutual predictions between body pain and mental fatigue over the one-year interval were not significant. Additionally, gender had significant effects on body pain, physical fatigue, and mental fatigue.

**Conclusion:**

In Chinese adolescents, body pain and physical fatigue predict each other over a one-year period, and so do physical fatigue and mental fatigue. Health interventions targeting adolescents should address both body pain and physical and mental fatigue simultaneously, with particular emphasis on female adolescents.

## 1 Introduction

Body pain and fatigue are common symptoms of subhealth among adolescents (1, 2). Body pain refers to transient and frequent pain caused by non-clinical diseases or injuries, often below the threshold for clinical treatment (3, 4). It is highly prevalent among adolescents. Among the global youth population, the prevalence of headache was 54.1%, stomachache 49.8%, backache 37%, and at least one of these three pains 74.4% (5). The incidences of neck-shoulder pain and low back pain in the Chinese adolescent population were 41.1 and 32.8%, respectively (6).

Fatigue, which can be primarily classified as mental and physical, is best defined as a condition characterized by a decline in the ability and efficiency of mental and/or physical activities. This decline is caused by excessive mental or physical exertion or illness. Fatigue is often accompanied by a distinctive sense of discomfort, a desire to rest, and reduced motivation, collectively referred to as fatigue sensation (7). Fatigue is a prevalent health issue among adolescents, particularly among Chinese youth facing intense academic competition (8, 9).

Body pain and fatigue share a common pathological mechanism: low-grade inflammation. One review indicates that chronic psychosocial stress (e.g., long-term academic pressure) induces prolonged activation of the hypothalamic–pituitary–adrenal (HPA) axis, leading to cortisol resistance and a sustained elevation of pro-inflammatory cytokines (e.g., IL-6, TNF-*α*, CRP). These inflammatory factors act on the central nervous system through the blood–brain barrier, inducing sickness behavior characterized by significant fatigue, hyperalgesia, and low mood. This “stress–inflammation–symptom” vicious cycle ultimately results in functional widespread pain and chronic fatigue (10). Further studies have shown that subclinical inflammation is related to increased pain sensitivity and lowered pain thresholds (11, 12). One study suggests that fatigue, increased pain sensitivity, and anhedonia are core features of low-grade inflammation-induced sickness behavior (13).

Cross-lagged analyses have produced inconsistent findings regarding the relationship between body pain and fatigue. For example, one study involving community-dwelling adolescents found a significant bidirectional predictive relationship between changes in fatigue and levels of pain intensity and functional impairment (14). Another study reported that pain severity predicted subsequent physical fatigue; however, physical fatigue did not predict later pain severity (15). In a study of outpatients, baseline body pain significantly predict follow-up fatigue—which included physical fatigue, mental fatigue, and motivation—while baseline fatigue had no effect on follow-up body pain (16).

Furthermore, both mental fatigue, characterized by cognitive decline, and physical fatigue can result from low-grade inflammation. Persistent low-grade inflammation affects cerebral neurotransmitter metabolism, induces neuroinflammation, and damages reward circuits, simultaneously leading to physical exhaustion, psychological anhedonia, and cognitive decline (17, 18). One study demonstrated that mental fatigue can cause an earlier onset of physical fatigue during cycling tasks (19); another study found that, at the subjective level, the impact of physical fatigue on mental fatigue was greater than the reverse effect of mental fatigue on physical fatigue (20).

Two notable limitations of previous research necessitate the present study. First, few studies have distinguished between physical fatigue and mental fatigue; instead, they have either combined the two into a single indicator (16) or used physical fatigue as a proxy for the broader construct of fatigue (14). Second, previous findings are inconsistent. For example, one study found that fatigue and chronic pain could predict each other over time (14), whereas another study showed that pain severity had a unidirectional cross-temporal predictive effect on physical fatigue (15). Furthermore, given that body pain and fatigue are major symptoms of subhealth among Chinese adolescents, a thorough investigation of their specific interrelationship will help inform targeted intervention strategies.

Based on the literature review presented above, this study proposes three research hypotheses concerning Chinese adolescents:

*Hypothesis 1*: Body pain and physical fatigue predict each other over time.

*Hypothesis 2*: Body pain and mental fatigue predict each other over time.

Hypothesis 3: physical fatigue and mental fatigue predict each other over time.

Finally, research has shown that gender contributes to variations in pain experience (21), with girls exhibiting significantly higher fatigue scores than boys among children and adolescents (22). Therefore, this study take gender as a control variable.

## 2 Research methods

### 2.1 Sampling procedure

Students from two middle schools in Sichuan Province were selected as participants in this study using a convenience sampling method. A longitudinal tracking design was employed, with two waves of surveys conducted in April 2023 (Time 1) and April 2024 (Time 2). A total of 1,081 questionnaires were satisfactorily completed at Time 1 and included in the present study, while 966 questionnaires were satisfactorily completed in the second wave, resulting in a loss of 115 students, corresponding to a loss rate of 10.64%. The primary reasons for participant attrition at Time 2 included transfers, leaves of absence, and a high rate of incomplete questionnaire responses.

No significant differences were found between participants who completed the second survey (*n* = 966) and those lost to follow-up or with incomplete responses (*n* = 115) in gender distribution (*χ*^2^ = 0.18, df = 1, *p* > 0.05), Time 1 body pain (*t* = 0.124, df = 1,079, *p* > 0.05), physical fatigue (*t* = 0.970, df = 1,079, *p* > 0.05), or mental fatigue (*t* = 0.227, df = 1,079, *p* > 0.05). Thus, no systematic attrition was evident. The final sample comprised 966 students who completed all assessments; their baseline characteristics are shown in Table 1.

**Table 1:** Basic characteristics of the final sample (*N* = 966).

		Frequency	Percentage
Age (*M* = 13.78, SD = 0.81)	12	11	1.1
	13	386	40
	14	402	41.6
	15	146	15.1
	16	21	2.2
Gender	Male(=1)	474	49.1
	Female(=2)	492	50.9
Grade	7	225	23.3
	8	741	76.7
Family address	Village	209	21.6
	Town	322	33.3
	City	435	45.0
Ethnicity	*Han*	784	81.2
	Minority	182	18.8
Height (*M* = 160.38, SD = 8.12)	Male	163.41	8.85
	Female	157.47	6.05
Number of siblings	0	353	36.5
	1	240	24.8
	2	335	34.7
	3	35	3.6

### 2.2 Instruments

The five items for measuring body pain were derived from two previous studies (23, 24), assessing headache, stomach pain, neck and shoulder pain, back pain, and arm/leg pain. Participants recalled anybody pain experienced in the past month, excluding menstruation-, trauma-, or obvious illness-related pain, and rated each item on a five--point scale (0 = never, 1 = occasionally, 2 = sometimes, 3 = often, 4 = always). Higher mean scores indicate greater pain frequency.

Mental fatigue was measured using four items adapted from (39). Participants rated their mental status in class over the past month, including attention, memory, interest in courses, and clarity of thought (e.g., “I have difficulty concentrating in class”) (25).

Physical fatigue was measured using six items adapted from (39). Participants rated their physical status in daily life over the past month, including tiredness, lack of energy, sleepiness or drowsiness, muscle weakness, need for more rest, and easy fatigability during activities (e.g., “I need to rest more in daily life”) (25).

Demographic variables (age, gender, grade, family location, ethnicity, height, and number of siblings) were based on the second wave of surveys.

### 2.3 Ethics statement

Biomedical Research Involving Human Subjects. It was approved by the Academic Committee of Xichang University (Approval No. Xichang University 2,023,032). The research objectives, methods, potential risks, and benefits were thoroughly explained to the participants’ legal guardians, who then provided voluntary written informed consent.

### 2.4 Data processing methodology

First, Harman’s single-factor test was employed to assess common method bias. Next, descriptive statistics and Pearson correlation coefficients were calculated for the entire sample. Finally, saturated structural equation model was constructed using Mplus to test the research hypotheses.

## 3 Research results

### 3.1 Common method bias test

This study employed Harman’s single-factor test to assess common method bias across two waves of data collection. In Wave 1, the first factor accounted for 39.47% of the total variance; in Wave 2, it accounted for 39.49%. Both values fall below the 40% threshold, indicating that common method bias is not a significant concern in this study.

### 3.2 Descriptive statistics and correlation analysis

Table 2 presents the means, standard deviations, and correlation coefficients for the main variables.

**Table 2:** Descriptive data and Pearson correlations in the whole sample (*N* = 966).

		M	SD	1	2	3	4	5	6
1	Body pain T1	0.71	0.70	*0.781*					
2	Physical fatigue T1	1.38	1.05	0.603^**^	*0.848*				
3	Mental fatigue T1	1.37	0.95	0.353^**^	0.503^**^	*0.837*			
4	Body pain T2	0.73	0.73	0.596^**^	0.484^**^	0.286^**^	*0.823*		
5	Physical fatigue T2	1.20	1.05	0.479^**^	0.604^**^	0.390^**^	0.671^**^	*0.886*	
6	Mental fatigue T2	1.28	0.94	0.283^**^	0.400^**^	0.574^**^	0.465^**^	0.617^**^	*0.856*

Italicized numbers on the diagonal are Cronbach’s alpha coefficients. T1 = at Time 1 T2 = at Time 2. ^**^p < 0.01, ^*^p < 0.05.

As shown in Table 2, Cronbach’s alpha coefficients for bodily pain, physical fatigue, and mental fatigue across both waves were all above 0.78, indicating good scale reliability in this study.

According to a previous study (26), correlation coefficients between 0.10 and 0.39 indicate a weak correlation, and those between 0.40 and 0.69 indicate a moderate correlation. As shown in Table 2, baseline body pain was moderately correlated with body pain at Time 2 (*r* = 0.596) and physical fatigue at Time 2 (*r* = 0.479), but weakly correlated with mental fatigue at Time 2 (*r* = 0.283). Baseline physical fatigue was moderately correlated with body pain at Time 2 (*r* = 0.484), physical fatigue at Time 2 (*r* = 0.604), and mental fatigue at Time 2 (*r* = 0.400). Baseline mental fatigue was weakly correlated with body pain at Time 2 (*r* = 0.286) and physical fatigue at Time 2 (*r* = 0.390), but moderately correlated with mental fatigue at Time 2 (r = 0.574).

### 3.3 Saturated structural equation model

Using Mplus 7.0, a saturated model was constructed to test the three research hypotheses. The path coefficients for all models are presented in Table 3.

**Table 3:** Path coefficients of the three models.

Path	*β*	SE
Auto-regression		
Body pain T1 → Body pain T2	0.454^**^	0.034
Physical fatigue T1 → Physical fatigue T2	0.427^**^	0.035
Mental fatigue T1 → Mental fatigue T2	0.501^**^	0.035
Correlations at T1		
Body pain T1 ↔ Physical fatigue 1	0.581^**^	0.024
Body pain T1 ↔ Mental fatigue 1	0.347^**^	0.034
Physical fatigue T1 ↔ Mental fatigue 1	0.502^**^	0.029
Correlations at T2		
Body pain T2 ↔ Physical fatigue T2	0.512^**^	0.027
Body pain T2 ↔ Mental fatigue T2	0.376^**^	0.031
Physical fatigue T2 ↔ Mental fatigue T2	0.520^**^	0.027
Cross-lagged regression		
Body pain T1 → Physical fatigue T2	0.157^**^	0.033
Body pain T1 → Mental fatigue T2	0.020	0.036
Physical fatigue T1 → Body pain T2	0.155^**^	0.036
Physical fatigue T1 → Mental fatigue T2	0.124^**^	0.037
Mental fatigue T1 → Body pain T2	0.037	0.030
Mental fatigue T1 → Physical fatigue T2	0.111^**^	0.032
Effects of gender on T1		
Gender →Body pain T1	0.229^**^	0.031
Gender → Physical fatigue T1	0.236^**^	0.030
Gender → Mental fatigue T1	0.072^*^	0.032
Direct effects of gender on T2		
Gender → Body pain T2	0.155^**^	0.026
Gender → Physical fatigue T2	0.114^**^	0.026
Gender → Mental fatigue T2	0.052^†^	0.027

T1 = at Time 1, T2 = at Time 2. ^**^p < 0.01, ^*^p < 0.05. ^†^indicates *p* = 0.052.

As the model is a saturated structural equation model (i.e., with zero degrees of freedom), the key fit indices achieve perfect values: χ^2^ = 0, df = 0, CFI = 1.00, TLI = 1.00, RMSEA = 0.00, SRMR = 0.00.

As presented in Table 3, body pain at Time 1 has a positive effect on physical fatigue at Time 2 (*β* = 0.157, *p* < 0.01). Simultaneously, physical fatigue at Time 1 positively affects body pain at Time 2 (*β* = 0.155, *p* < 0.01). According to prior research, a standardized coefficient of approximately 0.15 indicates a medium effect. The bidirectional predictive effects between body pain and physical fatigue (*β* ≈ 0.16) both reached the medium level, suggesting practically meaningful mutual predictions (27). Therefore, hypothesis 1 is strongly supported, indicating that adolescent body pain and physical fatigue predict each other over time.

Regarding hypothesis 2, body pain at Time 1 does not have a significant effect on mental fatigue at Time 2 (*β* = 0.020, *p* > 0.05), and mental fatigue at Time 1 does not significantly predict body pain at Time 2 (*β* = 0.037, *p* > 0.05). Thus, hypothesis 2 is not supported, indicating that body pain and mental fatigue do not predict each other over time.

For hypothesis 3, physical fatigue at Time 1 has a positive effect on mental fatigue at Time 2 (*β* = 0.124, *p* < 0.01), and mental fatigue at Time 1 also positively predicts physical fatigue at Time 2 (*β* = 0.111, *p* < 0.01). The bidirectional predictive effects between physical fatigue and mental fatigue (β ≈ 0.11–0.12) approached a medium effect; although slightly below 0.15, they still had practical significance. (27). Hence, hypothesis 3 is supported, indicating that physical fatigue and mental fatigue predict each other over time.

As shown in Table 3, gender significantly predicted all three indicators at T1 (*β* = 0.229 to 0.072, *p* < 0.05) and directly predicted T2 body pain and physical fatigue (*β* = 0.155 and 0.114, *p* < 0.01), with a marginal effect on T2 mental fatigue (*β* = 0.052, *p* = 0.052). These results indicate that female adolescents consistently reported higher levels of body pain and fatigue across both waves.

Gender accounted for 5.3, 5.5, and 0.5% of the variance in T1 body pain, physical fatigue, and mental fatigue, respectively. The full model explained 40.2, 40.6, and 34.9% of the variance in T2 body pain, physical fatigue, and mental fatigue, respectively. These results indicate that body pain and fatigue exhibit strong temporal stability and cross-lagged effects, and that gender plays a consistent role in their long-term prediction.

## 4 Discussion

### 4.1 The reciprocal relationships of body pain, physical fatigue, and mental fatigue in adolescents

Using longitudinal data, this study examined the relationship between body pain and fatigue in adolescents. Correlation analyses revealed that at two time points spanning 1 year, baseline body pain, physical fatigue, and mental fatigue were all significantly positively correlated with their respective follow-up measures as well as with each other, indicating that body pain and fatigue exhibit stable cross-temporal associations over a one-year period (see Table 2).

The results of cross-lagged analysis showed that Hypothesis 1 was strongly supported, indicating that body pain and physical fatigue predict each other over time (see Table 3). This finding aligns with the results of a previous study by Sommer et al. (14), which demonstrated that physical fatigue and somatic pain intensity mutually predict each other over time among children and adolescents. However, the findings of this study contradict those of another prior study, which reported that baseline body pain has a unidirectional predictive effect on subsequent physical fatigue (15, 16). The discrepancy may be attributed to differences in study populations: the present study and Sommer et al. (14) examined healthy adolescents, whereas Yamada et al. (15) and Ranum et al. (16) focused on clinical patients with primary pain symptoms.

The results indicate that body pain and mental fatigue did not significantly predict each other over the one-year interval (see Table 3); therefore, Hypothesis 2 was not supported. This finding contradicts the conclusions of previous studies. For instance, two earlier studies indicated that body pain predicts subsequent mental fatigue in patients with clinical chronic pain (28, 29), and an experiment revealed that cognitive fatigue can enhance experimentally induced pain perception by diminishing one’s ability to distract from discomfort (30). The discrepancy in conclusions may stem from the fact that healthy adolescents have only mild low-grade inflammation, which is insufficient to establish a stable cross-temporal association between body pain and mental fatigue. Moreover, the pain experienced by participants in this study was not experimentally manipulated.

Hypothesis 3—that physical fatigue and mental fatigue predict each other over time—was strongly supported (see Table 3). This finding is similar to those of previous studies (19, 20). For example, an experimental study found that, at the subjective level, physical fatigue and mental fatigue mutually influence each other, with the effect of physical fatigue on mental fatigue being stronger than the reverse (20).

In summary, this study found that body pain and physical fatigue, as well as physical fatigue and mental fatigue, mutually predicted each other over a one-year period, whereas no such cross-temporal relationship existed between body pain and mental fatigue. This indicates that, although body pain, physical fatigue, and mental fatigue can all be triggered by low-grade inflammation, their cross-temporal relationships are not identical over time.

### 4.2 The effects of gender on body pain, physical fatigue, and mental fatigue

This study found that gender has significant effects on body pain, physical fatigue, and mental fatigue, suggesting that compared to male adolescents, female adolescents experienced significantly higher levels of body pain, physical fatigue, and mental fatigue. These findings are consistent with previous research. For instance, a prior study reported that adolescent females experienced more pain than their male counterparts (31). Another study found that among German adolescents, females reported higher levels of both physical and psychological fatigue (14). The reason for these gender differences may primarily lie in the fact that, in adolescents, low-grade inflammation levels are significantly higher in females than in males (32).

### 4.3 Implications

This study examined the reciprocal relationships between body pain, physical fatigue, and mental fatigue in adolescents using longitudinal data. Theoretically, these findings enhance our understanding of the specific cross-temporal relationships between body pain and different types of fatigue. Although body pain, physical fatigue, and mental fatigue can all be triggered by low-grade inflammation, the present study demonstrates that over a one-year interval, body pain and physical fatigue predict each other, and physical fatigue and mental fatigue predict each other, whereas body pain and mental fatigue do not. This suggests that while body pain and fatigue share common physiological processes, they exhibit distinct patterns of temporal association: somatic symptoms (body pain and physical fatigue) are consistently associated with each other, and fatigue-related symptoms (physical fatigue and mental fatigue) are also consistently associated with each other, but cross-category associations (body pain and mental fatigue) are absent.

The results of this study highlight two aspects of practical significance. First, interventions targeting adolescent health should consider the reciprocal relationship between physical and psychological symptoms. As core components of suboptimal health status, body pain and physical fatigue are bidirectionally related over time, as are physical fatigue and mental fatigue. Therefore, a combination of psychological counseling and physiotherapy techniques (e.g., auricular acupressure and Tai Chi) should be implemented in school settings (33–35).

Second, gender-specific strategies are needed to address body pain and fatigue. The present study shows that female adolescents report higher levels of both compared to their male counterparts, so interventions should prioritize their needs. For example, since female adolescents tend to exert greater academic effort and academic stress significantly contributes to fatigue and body pain, academic stress management strategies should be specifically tailored for them (36).

### 4.4 Strengths and limitations

This study has two major strengths. First, it utilizes longitudinal (rather than cross-sectional) data to examine the bidirectional and predictive relationships between body pain, physical fatigue, and mental fatigue among Chinese adolescents, thereby informing health management strategies for this population. Second, by distinguishing between physical and mental components of fatigue and incorporating both into the model, the study offers a more nuanced understanding of the relationship between body pain and fatigue.

However, this study has several potential limitations. First, the one-year data collection interval may be insufficient to capture long-term effects, as fatigue has been shown to predict the onset of chronic widespread pain in adults over periods ranging from 5 to 18 years (37). Second, data were collected from only two middle schools in Sichuan Province, limiting the generalizability of our findings to the broader Chinese adolescent population, given substantial regional variations in basic education. Third, this study did not report student background information closely related to body pain and fatigue, such as academic stress and socioeconomic status, which limits a deeper interpretation of the findings. Lastly, body pain was assessed using only five items covering five locations, whereas adolescent body pain may occur in many other areas (38), potentially compromising measurement validity. Future research should address these gaps and further investigate the relationship between body pain and fatigue.

## 5 Conclusion

This study analyzed the relationship between body pain and fatigue in adolescents based on longitudinal data. The results showed that body pain and physical fatigue mutually predict each other over a one-year interval, and physical fatigue and mental fatigue also exhibit a bidirectional predictive relationship over time. However, the mutual predictions between body pain and mental fatigue over the one-year interval were not significant. Additionally, female adolescents report higher levels of body pain, physical fatigue, and mental fatigue compared to their male counterparts. Therefore, adolescent health interventions should address both body pain and physical and mental fatigue, with particular emphasis on female adolescents.

## Data Availability

The raw data supporting the conclusions of this article will be made available by the authors, without undue reservation.
